# Seasonal dynamics of pheromone traps and lures to monitor stink bugs (Hemiptera: Pentatomidae) in soybean

**DOI:** 10.1093/jee/toag125

**Published:** 2026-05-11

**Authors:** Taynara Possebom, Dominic Duane Reisig

**Affiliations:** Department of Entomology and Plant Pathology, North Carolina State University, Raleigh, NC, USA; Department of Entomology and Plant Pathology, North Carolina State University, Plymouth, NC, USA

**Keywords:** Semiochemicals, IPM, Sampling, Pentatomidae, Hemiptera

## Abstract

Stink bugs (Hemiptera: Pentatomidae) are major pests of soybean in the southeastern United States, causing $279 million in soybean costs and losses over the past 6 years. Current field-based direct scouting techniques could be supported by indirect monitoring systems. This study evaluated the effectiveness of seven pheromone trap types (delta, black pyramid, yellow pyramid, blue, clear, yellow, and white sticky cards) and two lure types/formulation to capture stink bugs across 68 soybean fields. From August to December 2023 and 2024, we checked the traps twice a month, counted stink bugs, identified stink bug species, and recorded trap captures at each soybean growth stage. Stink bug abundance varied by trap and lure combination. Pyramid traps captured the greatest species diversity. Trap captures also varied across soybean reproductive stages, with species-specific peaks occurring from flowering (R1) to maturity (R7). The dual-component lure (murgantiol <1%, methyl E, E, Z-2,4,6-decatrienoate <1%) consistently attracted more stink bugs than the single-component lure (methyl 2-E, 4Z-decadienoate <1%). These findings demonstrate that ground-deployed pyramid traps combined with dual-component lures may be effective tools for monitoring economically important stink bug species. Future research should explore the correlation between trap captures and in-field stink bug densities.

## Introduction

Stink bugs (Hemiptera: Pentatomidae) are a major pest of cultivated crops such as cotton (*Gossypium hirsutum* L.), corn (*Zea mays* L.), and soybean (*Glycine max* (L.) Merrill) (Negrón and Riley 1987, Panizzi 1997, Greene et al. 2001, Toews et al. 2008, Reay-Jones 2010, Koch and Rich 2015, Panizzi et al. 2018). They feed by inserting their stylet and injecting digestive saliva. This liquefies plant tissue, resulting in malformed seeds and fruits and sometimes reducing yield (McPherson and McPherson 2000).

In row crops, stink bug infestations can be particularly challenging because they involve multiple species with differing biology, host preferences, phenology, and injury patterns, often occurring simultaneously and across overlapping generations. These factors complicate management, especially in regions with diverse cropping systems like the southeastern US, where multiple stink bug species occur throughout the growing season, including southern green stink bug (*Nezara viridula* L.), rice stink bug (*Oebalus pugnax* Fabricius), brown marmorated stink bug (*Halyomorpha halys* Stål), green stink bug (*Chinavia halaris* Say), and brown stink bug (*Euschistus* spp. Say) (Bryant 2020). These species are classified as the stink bug complex in the southeastern US row crop system. Importantly, species within this complex differ in seasonal timing (Herbert and Toews 2011), movement among host plants (Jones and Sullivan 1982), susceptibility to insecticides (Panta et al. 2026a), and the type and severity of crop injury they cause (Jensen and Newsom 1972, Willrich et al. 2004), limiting the effectiveness of uniform management strategies. Among these, the brown stink bug (*Euschistus* spp., which represents a species complex) is particularly notable for its species diversity and abundance. In North Carolina and Virginia, this genus includes *Euschistus servus* (Say), *Euschistus tristigmus tristigmus* (Say), *Euschistus servus euschistoides* (Vollenhoven), *Euschistus conspersus* Uhler*, Euschistus ictericus* (L.), *Euschistus obscurus* (Palisot), *Euschistus crassus* (Dallas), *Euschistus tristigmus luridis* (Dallas), *Euschistus quadrator* Rolston, *Euschistus variolarius* (Palisot), and *Euschistus politus* Uhler (Panta et al. 2026b).

From 2019 to 2024, the stink bug species complex was the most economically damaging soybean insect pest across 18 US states, infesting nearly 62% of acres and with more than 15% of all fields exceeding economic thresholds. During this period, average annual costs and yield losses reached ∼$279 million (Musser et al. 2020, Musser et al. 2021, Musser et al. 2022, 2023, Musser et al., 2024, 2025). A key factor driving these losses is the timing of stink bug infestation relative to soybean reproductive development. Stink bugs are a mid- to late-season soybean pest, typically appearing after flowering and remaining until harvest, as developing pods and seeds make the plant more attractive for feeding and reproduction (Todd and Herzog 1980, Schumann and Todd 1982, Koch and Pahs 2014, Eduardo et al. 2018, Pezzini et al. 2019, Defensor et al. 2020).

Stink bug management in soybean relies on region-specific threshold recommendations that vary depending on the scouting technique (McPherson et al. 1993, Greene et al. 2001, Aigner et al. 2015). In North Carolina, beat cloth thresholds are set for relatively wide row widths (76.2 to 101.6 cm), which is 3.28 stink bugs per row-meter. An additional threshold is used for the sweep net of 5 stink bugs per 15 sweeps (Owens 2012), which is one of the most widely used sampling methods. These thresholds are typically doubled once soybeans reach growth stage R6.5 (Reisig 2021). Soybean stink bug thresholds vary little across states. In Louisiana and Minnesota, for example, the threshold is close to that of North Carolina, ranging from 9 to 10 stink bugs per 25 sweeps (Villegas 2023, Koch 2024). In Ohio, the threshold is comparable, with recommendations of 4 stink bugs per 10 sweeps (Michel et al. 2015), which is equivalent to 10 per 25 sweeps.

In addition to direct threshold-based methods, indirect tools such as pheromone traps may detect stink bug presence before field scouting begins. Traditional scouting methods, including sweep nets and drop cloths, are effective for estimating in-field population densities, but are labor-intensive and require frequent sampling across large areas. In contrast, traps provide continuous, passive monitoring of stink bug presence over time, which may offer an early indication of immigration or population buildup. As a result, trap captures could be used to track stink bug phenology and scouting efforts spatially and temporally rather than replace conventional sampling. Although no economic thresholds exist in row crops based on trap captures, these tools have the potential to support traditional scouting techniques. However, a clear understanding of trap design, placement, and capture-density relationships is required before trap-based thresholds can be developed.

Previous studies in the United States based on pheromone monitoring have primarily focused on the brown marmorated stink bug, yet recent surveys reveal significant shifts in stink bug community composition in southeastern soybeans. In North Carolina and Virginia, four major groups now dominate the stink bug complex: green stink bug (29.72% of total abundance), brown marmorated stink bug (28.05%), southern green stink bug (20.07%), and the brown stink bug complex (16.10%), collectively accounting for over 93% of stink bug populations (Panta et al. 2026b). In addition, most research on pheromone traps for stink bugs has focused on specialty cropping systems, particularly fruit crops and associated non-crop host plants, rather than row crops (Leskey et al. 2012, Weber et al. 2014, Leskey et al. 2015, Morrison et al. 2016, Acebes-Doria et al. 2017, Rice et al. 2018). One study found that modified pyramid and pipe traps captured more brown marmorated stink bugs than delta or sticky cards (Rice et al. 2018). However, these trap modifications were only evaluated for this single species and did not address the broader stink bug complex. Moreover, while the modified traps showed promising results, they are not currently commercially available, limiting their practical use.

Several traps are currently available, including pyramid traps (Mizell and Tedders 1995), delta traps, and several colors of sticky cards. These traps vary in design and capture mechanism, which may influence their effectiveness across stink bug species and cropping systems. However, commercially available trap and lure combinations have not been systematically evaluated for their effectiveness in capturing a variety of stink bug species in row crops. Validating these tools across a wider range of species and cropping systems could support IPM strategies by improving the efficiency and precision of pest detection.

The objectives of our study were to (i) determine whether trap designs differed in their ability to capture stink bug species in soybean, and (ii) assess whether commercially available pheromone lure type/formulations varied in their attraction to specific stink bug species across soybean growth stages. We hypothesized that stink bug species composition and abundance would differ among trap and lure combinations. Specifically, we hypothesized that pyramid traps would capture a greater diversity and abundance of stink bug species compared to other trap types due to their larger surface area, ground-level deployment, and established efficacy for stink bug capture. Additionally, we hypothesized that pheromone trap catches would be correlated with certain soybean growth stages, reflecting known seasonal changes in stink bug movement and host use (Herbert and Toews 2011).

## Materials and Methods

### Data Collection

**Fig. 1.**
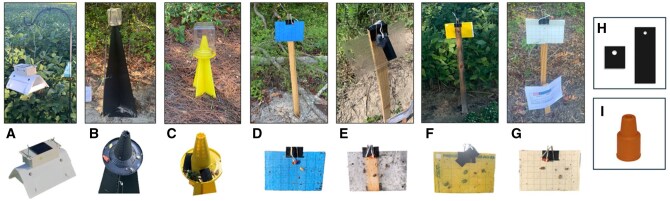
Trap types and pheromone lure type/formulation used in our experiment. (A) Delta trap, (B) Dead-Inn^TM^ black pyramid trap, (C) Dead-Inn^TM^ yellow pyramid trap, (D) blue sticky card, (E) clear sticky card, (F) yellow sticky card, (G) white sticky card, (H) dual commercial lure (murgantiol + methyl E, E, Z-2,4,6-decatrienoate), and (I) single commercial lure (methyl 2-E, 4Z-decadienoate).

We selected 68 soybean fields in Johnston County, North Carolina, United States, during 2023 and 2024. Fields were chosen based on a documented history of stink bug infestations in soybeans, as recorded by the local Cooperative Extension agent through grower-reported observations and scouting records from previous seasons. From this pool of fields with confirmed infestation history, fields were randomly selected using a random number generator, with each field serving as an independent experimental replicate. Along each field edge, we deployed seven different trap types spaced 50 m apart. Trap spacing was based on published effective range estimates of 70 to 130 m for brown marmorated stink bug pheromone-baited traps (Kirkpatrick et al. 2019), following methods from Rice et al. (2018). The seven trap types included: a Delta trap (FMC Corporation, Philadelphia, PA, United States; 27 cm base width × 12 cm height; Fig. 1A), Dead-Inn black pyramid trap (AgBio Inc., Westminster, CO, United States; 50.8 cm base width × 121.9 cm height; Fig. 1B), Dead-Inn yellow pyramid trap (AgBio Inc.,15.2 cm base width × 40.6 cm height; Fig. 1C), blue sticky card (Arbico Organics, Tucson, AZ, United States; 12.7 cm width × 17.8 cm length; Fig. 1D), clear sticky card (Trécé Inc., Adair, OK, United States; 13.5 cm in width × 25.5 cm in length; Fig. 1E), yellow sticky card (Trécé Inc., 13.5 cm width × 22.5 cm length; Fig. 1F), and white sticky card (Trécé Inc., 20.6 cm width × 20.6 cm length; Fig. 1G). Although the sticky cards came in different sizes, we standardized them in the laboratory by resizing all cards to 12.7 cm width × 17.8 cm. White sticky cards used inside the delta traps did not require resizing as they were designed to fit within the traps.

Prior to installing traps, we removed weeds from a 0.5 m radius around each trap location using a garden hoe. We attached each sticky card on top of a wooden stick (2.54 cm^2^ in width × 91.44 cm in height) using a black clip (5.08 cm in width × 8.13 cm in length). We secured the Delta trap with plastic twist ties and nylon cable ties (10 cm in width × 0.30 cm in length) to garden shepherd hooks (100 cm in height). We secured the black pyramid trap to the ground using four 100 cm steel stakes attached with the same nylon cable ties. We secured the yellow pyramid trap to the ground using four 40 cm steel stakes.

In half of the fields (selected at random), we placed a dual commercial lure type/formulation (Fig. 1H) consisting of two black rubber septa (one square and one rectangular) baited with a mixture of murgantiol (<1%) and methyl E, E, Z-2,4,6-decatrienoate (<1%) (Trécé Inc.), which is marketed to target green, southern green, and brown marmorated stink bugs. In the other half of the fields, we placed a single commercial lure type/formulation (Fig. 1I) consisting of an orange rubber cone baited with methyl 2-E, 4Z-decadienoate (<1%) (Trécé Inc.), which is marketed to target the brown stink bug complex. All traps were baited with at least one pheromone lure; no unbaited sticky traps were used. Although these lures are designed for specific species, we observed that they attracted other species, such as the harlequin bug (*Murgantia histrionica* Hahn), rice stink bug, red-shouldered stink bug (*Thyanta custator* Fabricius), and spine-shouldered stink bug (Podisus maculiventris Say).

We stored our pheromone lures at −20 °C and wore gloves when handling them in the field and laboratory to avoid cross-contamination, disposing of gloves between handling different lure types. We replaced the pheromone lures every 4 wk and checked the traps, replacing the sticky cards twice a month. We also evenly applied an additional sticky glue (Trécé Adhesives Division, Salinas, CA, United States) coat to the sticky cards because the original card adhesive was insufficient to retain stink bugs (they were still able to walk on the surface and escape). We used a spatula to apply the glue to the sticky cards on the same day that we replaced the traps. Finally, we recorded the number of nymphs and adults captured for the previously mentioned species from August to December during both years.

### Data Analysis

Before conducting statistical analyses, we divided our data into soybean growth stage periods. We conducted separate analyses for the full season (R1 to harvest; see Supplementary Tables and Figure) and for three sequential sub-periods analyzed together: beginning flowering to full pod (R1 to R4), full pod to beginning maturity (R4 to R7), and beginning maturity to harvest (R7 to harvest). Growth stages were pooled within each sub-period to minimize excess zeros associated with early- and late-season sampling (eg instead of analyzing R1 alone, we combined it with subsequent stages as R1 to R4). This approach improved our model performance by aligning trap captures with periods of biologically relevant stink bug activity.

We then evaluated the effects of trap type and lure type/formulation on stink bug capture. We analyzed the data and fitted a negative binomial generalized linear mixed model with a log link function to account for overdispersion that is typical for count data by using the glmmTMB package (Brooks et al. 2017, 2023), followed by ANOVA using the car package (Fox and Weisberg 2019). We conducted two levels of analysis: separate analyses for individual species, and an analysis of total captures combined across all species.

For individual species analyses, we examined the brown stink bug complex (*Euschistus* spp., Panta et al. 2026b), and four extra stink bug species: green stink bug, brown marmorated stink bug, southern green stink bug, and harlequin bug. Other stink bug species (rice stink bug, red-shouldered stink bug, and spined shouldered stink bug) were included in total capture counts but excluded from separate species-level analyses due to extremely low capture rates, providing a reliable statistical comparison. Even when we combined all other species, 94% of captures contained zero individuals from these species. For the analysis of total captures, the high frequency of zero captures across trap-growth stage combinations limited our ability to fit complex models with higher-order interactions. Therefore, we focused on the biologically meaningful two-way interaction of trap and lure.

Statistical analyses were conducted using RStudio version 1.2.5042 (RStudio Core Team 2020). Our dependent variable was the total number of stink bugs (combining adult and nymph captures) for each species or group of species. We included trap type, lure type/formulation, their interaction (trap × lure), soybean growth stage (R1 to R4, R4 to R7, R7 to harvest), and year (2023 and 2024) as fixed effects. We included the trap × lure interaction to determine if certain trap-lure combinations were more effective than others. For the harlequin bug, zero inflation prevented testing the trap × lure interaction; thus, only main effects are reported. We included fields as a random effect to account for spatial variation and the non-independence of traps within the same location. Because field numbers were reused across lure treatments, each unique field was identified by the combination of year, field number, and lure type/formulation. Thus, fields assigned the same numeric identifier, but different lure treatments represented different physical locations. Using this definition, the study included 68 unique field locations across the 2-year study (17 fields × 2 lure type/formulations × 2 years). We used the nbinom1 family specification, which assumes the variance increases linearly with the mean. For species where the trap × lure interaction was significant, we extracted estimated marginal means for each trap-lure combination within each growth stage period using the emmeans package. Pairwise comparisons of all trap-lure combinations within each period were conducted using compact letter displays with Sidak adjustment for multiple comparisons (*α* = 0.05). The Sidak method was applied because comparisons were conducted separately within each growth stage period (eg multiple families of comparisons). For species where the interaction was not significant, pairwise comparisons were conducted on main effects (trap type or lure type/formulation) using the same procedure. We then back-transformed estimated marginal means and model predictions from the log scale to the original count scale for visualization and interpretation. Finally, although the model estimated expected counts for each combination of trap type, lure type/formulation, and soybean growth stage, we present these values in the plots as back-transformed, model-estimated, average captures per trap sampling event to facilitate interpretation. These adjusted means account for variation due to field-level random effects, growth stage differences, and overdispersion in the count data.

## Results

### *Euschistus* spp. - Brown Stink Bug Complex

**Fig. 2.**
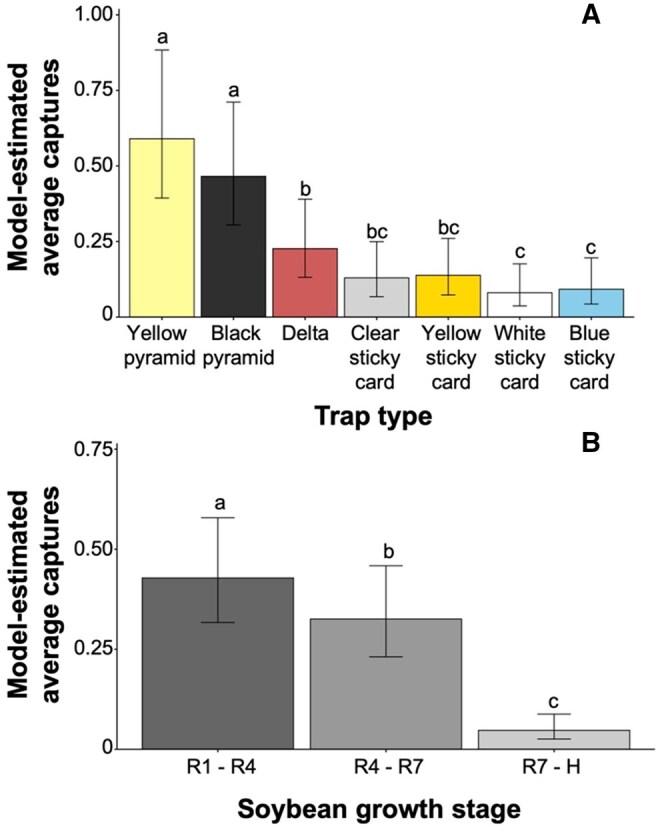
Mean (±SE) model-estimated average of brown stink bug complex captures by (A) trap type, and (B) soybean growth stage periods. Model-estimated captures represent predicted average counts per trap-lure combination per sampling event. Error bars represent the 95% CI. Pairwise comparisons used the Sidak test; trap × lure interaction was not significant (*P* > 0.05; Table 1). Bars that share the same letter are not significantly different based on multiple comparisons (*α* = 0.05).

We did not observe a significant interaction between traps and lure type/formulation on the average biweekly (twice a month) captures of the brown stink bug complex (Table 1), indicating that the effects of traps and lure type/formulation were independent. However, trap type had a significant effect on captures (Table 1), with both pyramid traps capturing significantly more brown stink bug complex than all other trap types (Fig. 2A). Additionally, the Delta trap captured more brown stink bug complex than the blue and white sticky cards, but did not differ significantly from the clear and yellow sticky cards (Fig. 2A).

**Table 1.** Analysis of variance results for the effects of trap type, lure type/formulation, and soybean growth stage (R1 to R4, R4 to R7, and R7 to harvest) on stink bug captures for each species

Dependent variable (number of stink bugs captured)	Independent variables	df	*χ* ^2^	*P*-value
***Euschistus* spp.** **brown stink bug complex**	Trap type (Tt)	6	74.83	<0.001
	Lure type/formulation (Ltf)	1	0.05	0.816
	Growth Stage	2	78.70	<0.001
	Year	1	0.77	0.378
	Tt Ltf	6	5.95	0.428
***Chinavia hilaris*** **green stink bug**	Tt	6	59.02	<0.001
	Ltf	1	27.34	<0.001
	Growth Stage	2	104.59	<0.001
	Year	1	2.36	0.123
	Tt Ltf	6	3.42	0.754
***Halyomorpha halys*** **brown marmorated stink bug**	Tt	6	169.47	<0.001
	Ltf	1	95.14	<0.001
	Growth Stage	2	174.30	<0.001
	Year	1	13.78	0.0002
	Tt Ltf	6	11.49	0.0741
***Nezara viridula*** **southern green stink bug**	Tt	6	79.18	<0.001
	Ltf	1	29.07	<0.001
	Growth Stage	2	65.88	<0.001
	Year	1	1.20	0.272
	Tt Ltf	6	8.67	0.192
***Murgantia histrionica*** **harlequin bug**	Tt	6	87.71	<0.001
	Ltf	1	97.85	<0.001
	Growth Stage	2	70.78	<0.001
	Year	1	3.97	0.040
	Tt Ltf	-	-	-
**Total captures**	Tt	6	206.52	<0.001
	Ltf	1	109.21	<0.001
	Growth Stage	2	270.82	<0.001
	Year	1	15.90	0.0004
	Tt Ltf	6	29.68	<0.001

The table presents degrees of freedom (df), Chi-square test statistics (*χ*^2^), and associated *P*-values for each main effect and the interaction between trap and lure type/formulation (Tt × Ltf). Significance levels: ***P ≤ 0.001, *P ≤ 0.05, not significant (*P* ≥ 0.05).

Lure type/formulation alone had no significant effect on stink bug capture (Table 1). In contrast, soybean growth stage had a significant effect (Table 1), with the highest average captures of brown stink bug complex from soybean growth R1 to R4, R4 to R7, and R7 to harvest sampling periods (Fig. 2B). Captures did not differ significantly between years (Table 1).

Complete full-season results (R1 to harvest) are presented in Supplementary Tables S1 and S2.

### *Chinavia hilaris* - Green Stink Bug

**Fig. 3.**
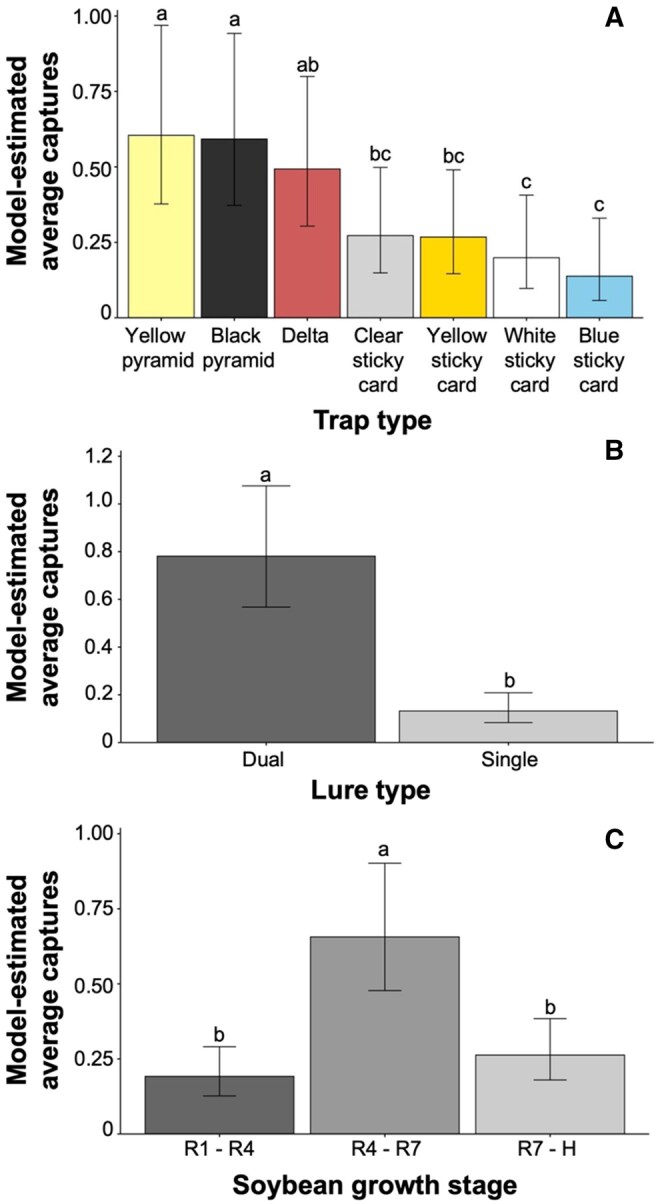
Mean (±SE) model-estimated average of *green stink bug* captures by (A) trap type, (B) lure type/formulation (single-component: methyl 2-E, 4Z-decadienoate; dual-component: murgantiol + methyl E, E, Z-2,4,6-decatrienoate), and (C) soybean growth stage period. Model-estimated captures represent predicted average counts per trap-lure combination per sampling event. Error bars represent the 95% CI. Pairwise comparisons used the Sidak test; trap × lure interaction was not significant (*P* > 0.05; Table 1). Bars that share the same letter are not significantly different based on multiple comparisons (*α* = 0.05).

We did not observe a significant interaction between traps and lure type/formulation on captures of green stink bugs (Table 1), indicating that the effects of traps and lure type/formulation were independent. However, trap type had a significant effect on captures (Table 1), with both pyramid traps capturing significantly more green stink bugs than all sticky cards, but did not differ significantly from the delta trap (Fig. 3A).

Lure type/formulation alone had a significant effect on stink bug captures (Table 1), with the dual lure attracting more green stink bugs compared to the single lure (Fig. 3B). Likewise, soybean growth stage had a significant effect (Table 1), with the highest average captures of green stink bug from soybean growth stages R4 to R7; the R1 to R4 and R7 to harvest sampling periods did not differ from each other (Fig. 3C). Captures did not differ significantly between years (Table 1).

Complete full-season results (R1 to harvest) are presented in Supplementary Tables S1 and S3.

### *Halyomorpha halys* - Brown Marmorated Stink Bug

**Fig. 4.**
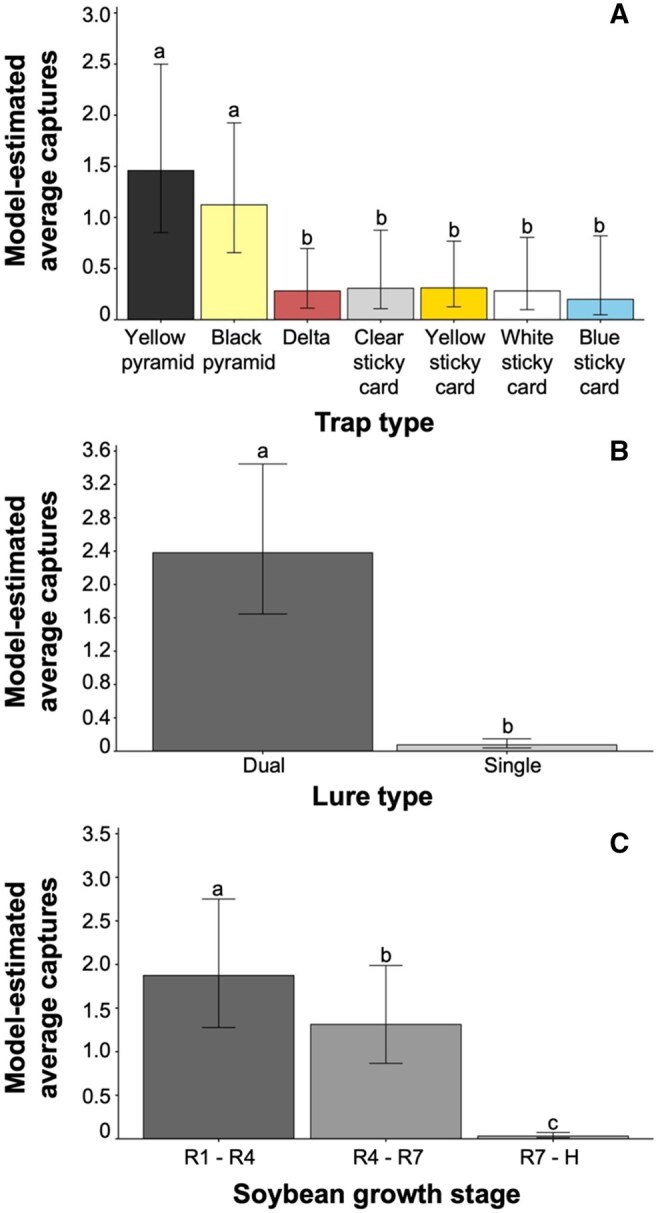
Mean (±SE) model-estimated average of brown marmorated *stink bug* captures by (A) trap type, (B) lure type/formulation (single-component: methyl 2-E, 4Z-decadienoate; dual-component: murgantiol + methyl E, E, Z-2,4,6-decatrienoate), and (C) soybean growth stage period. Model-estimated captures represent predicted average counts per trap-lure combination per sampling event. Error bars represent the 95% CI. Pairwise comparisons used the Sidak test; trap × lure interaction was not significant (*P* > 0.05; Table 1). Bars that share the same letter are not significantly different based on multiple comparisons (α = *0*.05).

We did not observe a significant interaction between traps and lure type/formulation on captures of brown marmorated stink bugs (Table 1), indicating that the effects of traps and lure type/formulation were independent. However, trap type had a significant effect on captures (Table 1), with both pyramid traps capturing significantly more brown marmorated stink bugs than all other trap types (Fig. 4A).

Lure type/formulation alone had a significant effect on stink bug captures (Table 1), with the dual lure attracting more brown marmorated stink bugs compared to the single lure (Fig. 4B). Likewise, soybean growth stage had a significant effect (Table 1), with the highest average captures of brown marmorated stink bug from soybean growth stages R1 to R4, R4 to R7, and R7 to harvest sampling periods (Fig. 4C). Captures differed significantly between years (Table 1), with approximately twice as many brown marmorated stink bugs captured in 2024 (0.62 ± 0.13 SE) than in 2023 (0.28 ± 0.06 SE).

Complete full-season results (R1 to harvest) are presented in Supplementary Tables S1 and S4.

### *Nezara viridula* - Southern Green Stink Bug

**Fig. 5.**
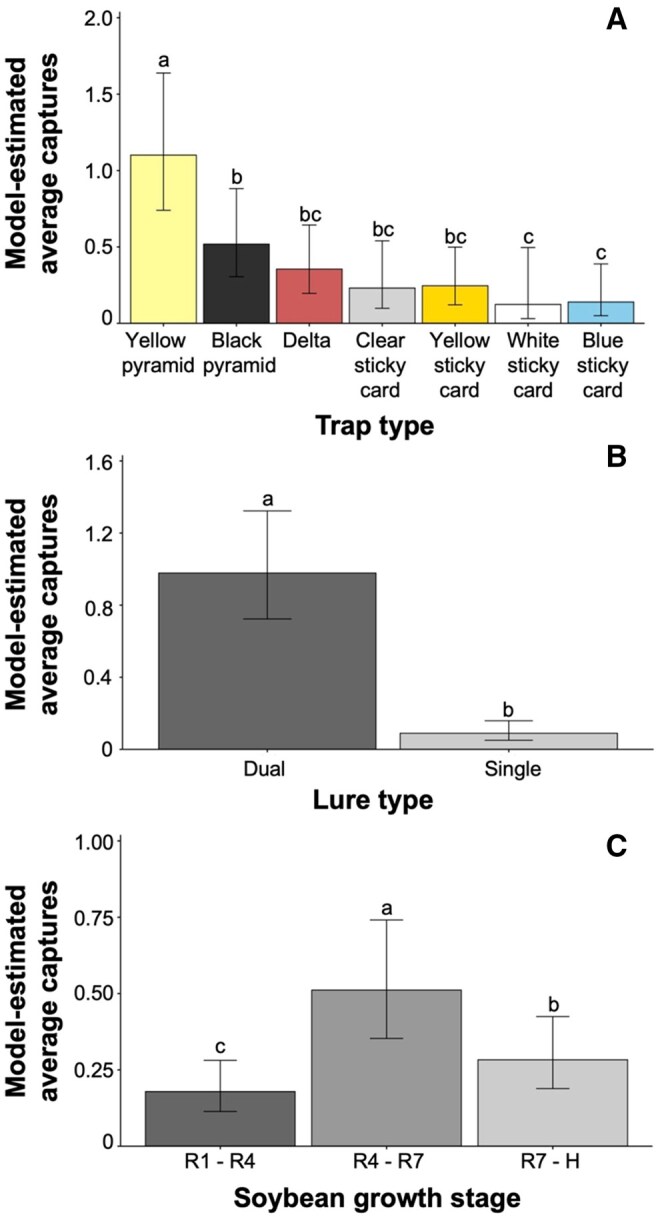
Mean (±SE) model-estimated average of southern green *stink bug* captures by (A) trap type, (B) lure type/formulation (single-component: methyl 2-E, 4Z-decadienoate; dual-component: murgantiol + methyl E, E, Z-2,4,6-decatrienoate), and (C) soybean growth stage period. Model-estimated captures represent predicted average counts per trap-lure combination per sampling event. Error bars represent the 95% CI. Pairwise comparisons used the Sidak test; trap × lure interaction was not significant (*P* > 0.05; Table 1). Bars that share the same letter are not significantly different based on multiple comparisons (α = *0*.05).

We did not observe a significant interaction between traps and lure type/formulation on captures of southern green stink bugs (Table 1), indicating that the effects of traps and lure type/formulation were independent. However, trap type had a significant effect on captures (Table 1), with the yellow pyramid trap capturing significantly more southern green stink bugs than all other trap types (Fig. 5A).

Lure type/formulation alone had a significant effect on stink bug captures (Table 1), with the dual lure attracting more southern green stink bugs compared to the single lure (Fig. 5B). Likewise, soybean growth stage had a significant effect (Table 1), with the highest average captures of southern green stink bug from soybean growth stages R4 to R7, R7 to harvest, and R1 to R4 sampling periods (Fig. 5C). Captures did not differ significantly between years (Table 1).

Complete full-season results (R1 to harvest) are presented in Supplementary Tables S1 and S5.

### *Murgantia histrionica* - Harlequin Bug

**Fig. 6.**
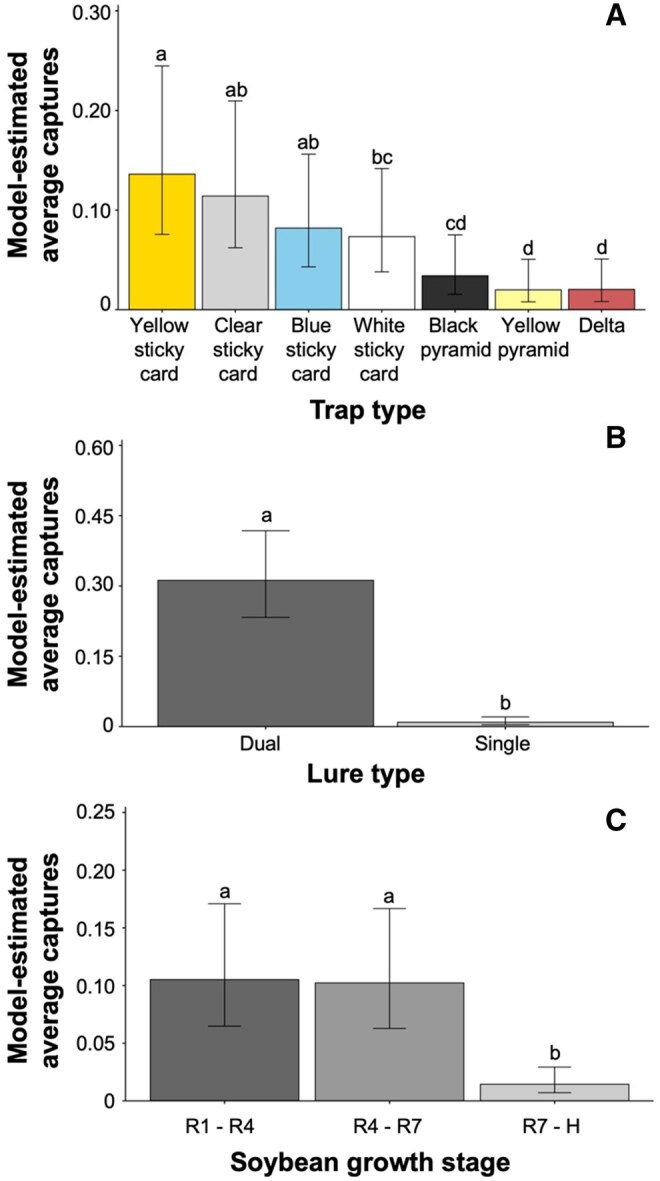
Mean (±SE) model-estimated average of harlequin bug captures by (A) trap type, (B) lure type/formulation (single-component: methyl 2-E, 4Z-decadienoate; dual-component: murgantiol + methyl E, E, Z-2,4,6-decatrienoate), and (C) soybean growth stage period. Model-estimated captures represent predicted average counts per trap-lure combination per sampling event. Error bars represent the 95% CI. Pairwise comparisons used the Sidak test; trap × lure interaction was not significant (*P* > 0.05; Table 1). Bars that share the same letter are not significantly different based on multiple comparisons (*α* = 0.05).

Due to zero inflation, we did not test the interaction between traps and lure type/formulation on captures of harlequin bugs; therefore, only the main effects are reported. Trap type had a significant effect on captures (Table 1), with the yellow, clear, and blue sticky cards capturing significantly more harlequin bugs than all other trap types, but clear and blue sticky cards did not differ significantly from white sticky cards (Fig. 6A). Lure type/formulation alone had a significant effect on harlequin bug captures (Table 1), with the dual lure attracting more harlequin bugs compared to the single lure (Fig. 6B). Likewise, soybean growth stage had a significant effect (Table 1), with similar average captures of harlequin bugs from soybean growth stages R1 to R4 and R4 to R7, followed by R7 to harvest sampling periods (Fig. 6C). Captures differed significantly between years (Table 1), with more harlequin bugs captured in 2023 (0.06 ± 0.01 SE) than in 2024 (0.04 ± 0.01 SE).

Complete full-season results (R1 to harvest) are presented in Supplementary Tables S1 and S6.

### Total Captures

**Fig. 7.**
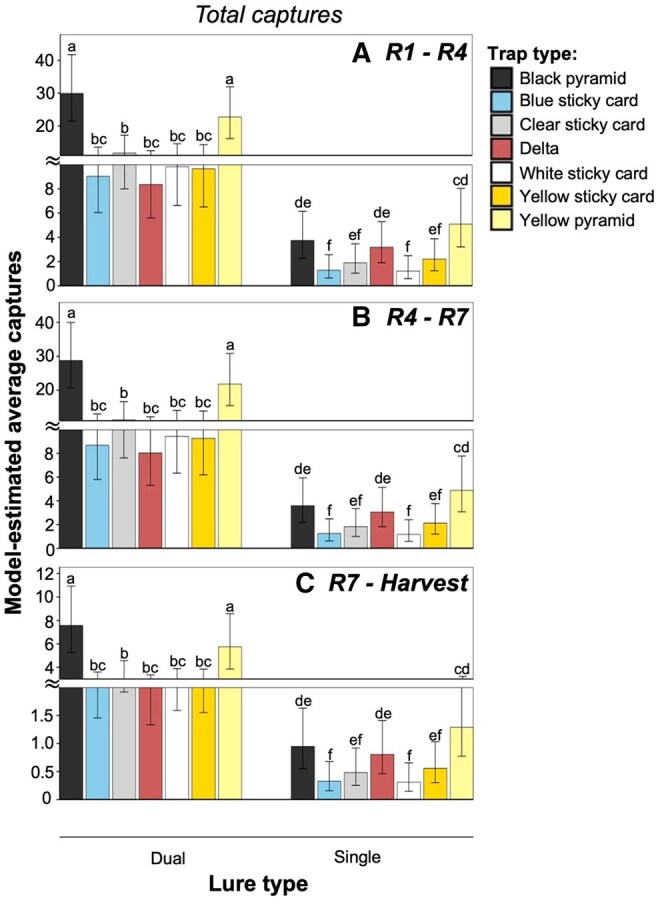
Mean (±SE) model-estimated average total captures (brown stink bug, green stink bug, brown marmorated stink bug, southern green stink bug, harlequin bug, rice stink bug, red-shouldered stink, and spine shouldered stink bug) by combinations of trap type and lure type/formulation (single-component: methyl 2-E, 4Z-decadienoate; dual-component: murgantiol + methyl E, E, Z-2,4,6-decatrienoate) across soybean growth stages: (A) beginning flowering to full pod (R4), (B) full pod to beginning maturity (R7), and (C) beginning maturity to harvest. Model-estimated captures represent predicted average counts per trap-lure combination per sampling event. Error bars represent the 95% CI. Pairwise comparisons used the Sidak test; trap × lure interaction was significant (*P* *<* 0.001; Table 1). Bars sharing the same letter within each panel are not significantly different (*α* = 0.05), based on multiple comparison tests. *Y*-axis scales differ between dual and single lure formulations; axis breaks (≈) indicate a change in scale.

We observed a significant interaction between traps and lure type/formulation on total stink bug captures (brown stink bug complex, green stink bug, brown marmorated stink bug, southern green stink bug, harlequin bug, rice stink bug, red-shouldered stink bug, and spine-shouldered stink bug; Table 1). Among all trap-lure combinations, pyramid traps paired with the dual lure consistently captured significantly more stink bugs (Fig. 7). This pattern remained consistent across all soybean growth stages, including R1 to R4 (Fig. 7A), R4 to R7 (Fig. 7B), and R7 to harvest (Fig. 7C).

Lure type/formulation and trap type alone had a significant effect on stink bug captures (Table 1), with the dual lure (8.00 ± 0.77) attracting more southern green stink bugs compared to the single lure (1.47 ± 0.17). Likewise, soybean growth stage had a significant effect (Table 1), with similar average captures of stink bug species from soybean growth stages R1 to R4 (5.50 ± 0.46) and R4 to R7 (5.28 ± 0.46), followed by R7 to harvest (1.39 ± 0.15) sampling periods. Captures differed significantly between years (Table 1), with more stink bugs captured in 2024 (4.51 ± 0.47 SE) than in 2023 (2.61 ± 0.28 SE).

Complete full-season results (R1 to harvest) are presented in Supplementary Tables S1 and Fig. S1.

## Discussion

Stink bug species abundance varied significantly with trap type and pheromone lure type/formulation combinations, with pyramid traps capturing the most species diversity, consistent with our hypotheses. Additionally, the dual lure consistently attracted more stink bug species than single lures. We also observed distinct peaks in activity at specific soybean growth stages that align with stink bug seasonal activity patterns. Brown stink bug complex and brown marmorated stink bug showed notably high captures during early reproductive stages (R1 to R4, beginning flowering to full pod), likely reflecting landscape-scale movement of bivoltine adults seeking food and reproductive hosts (Panizzi et al. 2000, Nielsen and Hamilton 2009, Goethe et al. 2021, Grabarczyk et al. 2022). While previous research suggested limited dispersal into soybean fields until R3 to R4 stages (Nielsen et al. 2011), our earlier trap captures possibly indicate that pheromone-baited traps detect initial field colonization before peak activity, which is critical since soybean plants are vulnerable to injury during both R4 and R6 stages (Owens et al. 2013, Koch and Rich 2015). Green stink bug and southern green stink bug peaked during mid reproductive stages (R4 to R7, full pod to beginning maturity), consistent with previous findings that southern green stink bug shows peak oviposition during pod fill and maximum densities during seed development (Schumann and Todd 1982). Both species show similar population patterns with increases in newly emerged adults near fall (Panizzi et al. 2000, Herbert and Toews 2012), but differ in voltinism: green stink bug is bivoltine while southern green stink bug is multivoltine (Ali et al. 1979, Jones and Sullivan 1982, Panizzi et al. 2000). This timing suggests that reproductive activity is synchronized with optimal host plant nutritional quality, as developing pods provide essential resources for egg production and nymph development (Possebom et al. 2020). Harlequin bug showed broader temporal presence from beginning flowering through beginning maturity, reflecting its multivoltine life cycle (3 to 4 generations in North Carolina) and cross-attraction to murgantiol, although it is not a soybean pest (Sullivan and Brett 1974, Zahn et al. 2008).

Complementing these seasonal patterns, our results also revealed important differences among trap designs in their ability to attract and capture different stink bug species. Overall, these findings indicate that we can use ground-deployed pyramid traps to capture various stink bug species in soybean, similar to other crops where various stink bug species are an economic pest (Mizell et al. 1996, Johnson et al. 2002, Leskey et al. 2012, Rice et al. 2018, Acebes-Doria et al. 2020). The only exception to the superior accuracy of pyramid traps was the harlequin bug, which was more effectively captured by yellow, clear, and blue sticky cards, reflecting its species-specific color preferences (DiMeglio et al. 2017). Although not a soybean pest, this finding could be valuable for monitoring in vegetable crops where harlequin bug is a pest (McPherson 1982, Capinera 2008, McPherson et al. 2018).

Beyond trap design, the effectiveness of pheromone-baited traps depends on understanding their ecological function in nature. Aggregation pheromones in stink bugs can serve multiple roles, including location of food sources, overwintering site selection, protection from parasites, and mate finding (Endo et al. 2006, Aldrich et al. 2007, Weber et al. 2018). The cross-species pheromone attraction observed in our study is not fully understood, but Weber et al. (2014) suggested that methyl (E, E, Z)-2,4,6-decatrienoate attraction in both adults and nymphs is primarily food-related, supporting prediapause feeding and overwintering site location.

Given this ecological context, lure type/formulation also had a significant effect on capture rates. Dual lures (murgantiol <1% and methyl E, E, Z-2,4,6-decatrienoate <1%, Trécé Inc.) consistently attracted more stink bugs than single lures (methyl 2-E, 4Z-decadienoate <1%, Trécé Inc.) across all species. This finding contrasts with the expectation that the single-component lure would be more effective for the brown stink bug complex, as it is specifically marketed for trapping this species. Tillman et al. (2010) also observed species-specific responses to pheromone blends in field conditions, including strong attraction of brown stink bug to methyl (E, Z)-2,4-decadienoate (Aldrich et al. 1991). However, our results suggest the commercial dual lure provided broader attraction or potential synergistic effects, resulting in higher captures, even for the brown stink bug complex. The exact cause of pheromone cross-attraction among Hemiptera species remains unknown (Tillman et al. 2010). One explanation is that aggregation may provide a passive defense against parasitism (Aldrich et al. 2007, Higaki and Adachi 2011).

This hypothesis has been supported across a range of animal taxa. In insects, *Malacosoma americanum* (gregarious eastern tent caterpillars) collectively thrash their bodies in coordinated group displays that actively deter parasitoid wasps and tachinid flies (Fitzgerald 1995). Similar patterns have been observed in vertebrates; for example, *Ovis canadensis mexicana* (desert bighorn sheep) displays fewer behaviors associated with biting fly harassment when bunching together (Mooring et al. 2003), further illustrating that aggregation as a defense against natural enemies is a broadly conserved strategy across the animal kingdom.

Another explanation is that stink bugs use pheromone cues to locate food sources (Krupke et al 2001, Endo et al. 2006, Aldrich et al. 2007, Grabarczyk et al. 2022). In our study, we frequently observed stink bugs on plants near pheromone-baited traps, a behavior also documented in previous studies (Aldrich et al. 1991, James et al. 1996, Krupke et al. 2001, Tillman and Cottrell 2019). Additionally, the pheromone release rate can influence whether individuals are attracted (aggregation) or repelled (dispersal), and this response may vary by instar or developmental stage (Ishiwatari 1974, 1976, Lockwood and Story 1986, Xu et al. 2025), which could support our observations with the single lure. Future research should explore whether blending lures could improve attraction or, conversely, create antagonistic effects among target species.

Additionally, stink bug captures varied by species and soybean reproductive growth stage, consistent with earlier literature findings that reported similar patterns across planting dates and maturity group (Newsom and Herzog 1977, Gore et al. 2006). These species-specific capture patterns likely reflect real biological processes, including colonization timing and the capacity of certain soybean stages (eg seed fill) to support successive stink bug generations, suggesting that trap catches are representative of population dynamics within the crop.

However, stink bug phenology in soybean does not occur in isolation. In the southeastern US, crop rotation provides a sequence of host plants, and the availability of adjacent crops strongly shapes stink bug distribution across the landscape (Russell 1952, Blinka 2008, Toews and Shurley 2009, Olson et al. 2012, Reisig et al. 2013). Previous studies have documented that species peaks in soybean often coincide with shifts from or into surrounding crops, highlighting how farmland scape composition interacts with soybean phenology to influence stink bug dynamics. For instance, the brown stink bug complex populations in soybean often peak when soybean blooms, while southern green stink bug populations peak near maturity, a fact supported by our trap captures.

Furthermore, stink bug movement into soybean from adjacent crops such as cotton, peanut, corn, and grain sorghum is not driven by a preference for soybean per se, but rather reflects a phenological response, as stink bugs relocate to whichever crop is at the most suitable reproductive stage for feeding and reproduction at a given time (Bundy and McPherson 2000, Smith et al. 2009, Toews and Shurley 2009, Olson et al. 2011).

This continuity of hosts can facilitate synchronized generations with seasonal crop cycles (Saulich and Musolin 2018) or trigger movement driven by changing nutritional needs (Panizzi and Slansky 1991, Panizzi and Parra 2012, Panizzi et al. 2018), plant phenology (Schumann and Todd 1982, Pezzini et al. 2019), and field edge environment (Fagan et al. 1999, Reay-Jones 2010, Reeves et al. 2010, Tillman 2011, 2014).

While our trap data reflect these biological and landscape patterns, because we sampled biweekly (twice a month), some individuals present between sampling dates may not have been recorded (Leskey and Hogmire 2005). This is because stink bugs may have escaped or been preyed upon between the biweekly sampling period. More frequent sampling could potentially capture finer-scale population dynamics. While the magnitude of this underestimation is unclear, these data still provide meaningful comparisons across species and soybean stages since all traps were sampled consistently. Other biotic and abiotic factors not captured in our studies may also influence trap counts and should be considered in future research. For example, parasitoids (Krupke and Brunner 2003, Tillman et al. 2023) attracted to stink bug pheromones could reduce local stink bug populations near traps, potentially affecting capture rates. Regional validation is also needed, as species composition and population phenology may vary across different geographic areas (Smith et al. 2009, Temple et al. 2013, Vyavhare et al. 2014, Pezzini et al. 2019). Finally, because both males and females were present in our trap collections and we combined adults and nymphs in our analyses, future studies should also evaluate potential differences in trap or lure attractiveness of the stink bug complex by sex or life stage to refine monitoring strategies.

Overall, our research showed that ground pyramid traps paired with multi-component lures represent a practical tool for tracking stink bug populations in soybean, consistently capturing greater species diversity and abundance than alternative trap-lure combinations. Our findings also revealed distinct species-specific peaks across soybean reproductive stages, the brown stink bug complex and brown marmorated stink bug during early pod development (R1 to R4), green stink bug and southern green stink bug during seed fill and maturation (R4 to R7). Although trap captures may not directly quantify immigration, emigration, or field densities, they reflect species-specific phenology and crop-stage associations that can inform management decisions.

To establish reliable relationships between trap catches and actual field populations, future research should integrate trapping data with direct in-field sampling methods. We propose studies that pair edge-based trap monitoring with in-field assessments (sweep net or beat sheet sampling, and visual plant counts) conducted at multiple distances from trap locations. Based on published effective range data for brown marmorated stink bug pheromone-baited traps, dispersive distances of 70 to 130 m (Kirkpatrick et al. 2019). We suggest sampling at distances of 25, 50, and 150 m from field edges to capture the gradient from within potential trap influence zones to areas likely beyond detection range, though optimal distances should be adjusted based on species-specific effective range data when available. Beyond species variation, sampling distances may vary depending on field size, crop type, and landscape context, as trapping area can be influenced by the presence of host plants and habitat structure. This spatial approach would reveal whether trap catches at field margins correlate with economically relevant in-crop densities throughout the field interior.

While direct sampling methods also have biases and detection limitations, they are also labor-intensive and costly. Traditional stink bug scouting requires an average of ∼35 min per soybean field (varying 0.3 to 120 ha) weekly throughout the growing season (Ribeiro et al. 2022), creating unsustainable labor demands for producers managing multiple fields. For example, a grower with only 10 fields would require ∼5 h per week for scouting alone, excluding travel time. Additionally, soybean growers report difficulties with sweep net sampling because it requires qualified workers and is very time-consuming when sampling large areas (Bueno et al. 2021).

In contrast, pheromone traps offer potential labor and cost savings through indirect monitoring that requires only weekly or biweekly trap checks. Comparing these complementary approaches (sweep net and pheromone traps) across multiple crop stages, sites, and seasons would establish whether consistent trap-to-density relationships exist. Such validation studies are essential to determine whether trap catches correlate with stink bug movement into and out of fields and with their actual densities within fields, a critical step before developing reliable economic thresholds. Previous research established a threshold of 10 brown marmorated stink bug adults per trap in apple orchards (Short et al. 2017), but further work is needed to refine and validate similar standards for soybean systems. Understanding these phenological patterns and their relationship to crop stage could provide a foundation for refining action thresholds and strengthening regionally tailored IPM programs.

## Supplementary Material

toag125_Supplementary_Data
