# Supplementary material for: Seasonal dynamics of pheromone traps and lures to monitor stink bugs (Hemiptera: Pentatomidae) in soybean
Source: J Econ Entomol. 2026 May 11;119(3):1919–30. doi: 10.1093/jee/toag125 (PMC13268538; doi:10.1093/jee/toag125)
Supplement: toag125_Supplementary_Data [file toag125_supplementary_data.zip › Supplementary Tables.docx]

**Table S1.** Analysis of variance (ANOVA) results for the effects of trap type and lure type/formulation*,* across full season soybean growth stage (beginning flowering (R1) to harvest) on stink bug captures for each species. The table presents degrees of freedom (Df), Chi-square test statistics (χ²), and associated P-values for each main effect and the interaction between trap and lure type/formulation (Tt × Ltf). Significance levels: P ≤ 0.001 ***, P ≤ 0.05 *, not significant (P ≥ 0.05). ^1^ For harlequin bug, zero inflation prevented testing the trap × lure interaction; thus, only main effects are reported.

| **Dependent variable** (Number of stink bugs captured) | **Independent**  **variables** | **Df** | ***χ ^2^*** | ***P*-value** |
| --- | --- | --- | --- | --- |
| Euschistus spp.  brown stink bug complex | Trap type (Tt) | 6 | 63.81 | < 0.001 *** |
|  | Lure type/formulation (Ltf) | 1 | 0.18 | 0.667 |
|  | Year | 1 | 1.57 | 0.210 |
|  | Tt * Ltf | 6 | 4.45 | 0.614 |
|  |  |  |  |  |
| Chinavia hilaris  green stink bug | Tt | 6 | 89.56 | < 0.001 *** |
|  | Ltf | 1 | 31.73 | < 0.001 *** |
|  | Year | 1 | 1.80 | 0.179 |
|  | Tt * Ltf | 6 | 3.83 | 0.699 |
|  |  |  |  |  |
| Halyomorpha halys  brown marmorated stink bug | Tt | 6 | 368.31 | < 0.001 *** |
|  | Ltf | 1 | 96.94 | < 0.001 *** |
|  | Year | 1 | 9.60 | 0.0019 ** |
|  | Tt * Ltf | 6 | 11.20 | 0.082 |
|  |  |  |  |  |
| Nezara viridula  southern green stink bug | Tt | 6 | 126.54 | < 0.001 *** |
|  | Ltf | 1 | 28.62 | < 0.001 *** |
|  | Year | 1 | 0.26 | 0.608 |
|  | Tt * Ltf | 6 | 5.28 | 0.507 |
|  |  |  |  |  |
| Murgantia histrionica  harlequin bug | Tt | 6 | 94.78 | < 0.001 *** |
|  | Ltf | 1 | 98.23 | < 0.001 *** |
|  | Year | 1 | 5.13 | 0.023 * |
|  | Tt * Ltf ^1^ | - | - | - |
|  |  |  |  |  |
| Total captures | Tt | 6 | 533.09 | < 0.001 *** |
|  | Ltf | 1 | 131.63 | < 0.001 *** |
|  | Year | 1 | 11.90 | 0.0004 *** |
|  | Tt * Ltf | 6 | 38.77 | < 0.001 *** |

**Table S2.** Model-estimated average of brown stink bug complex captures by the single main effects of trap type across full season soybean growth stage (beginning flowering (R1) to harvest). Model-estimated captures represent predicted average counts per sampling event. Mean and SE are on the response (count) scale. Pairwise comparisons used the Sidak adjustment. Levels sharing the same letter are not significantly different (α = 0.05).

| **Trap type** | | | |
| --- | --- | --- | --- |
| **Factor** | **Mean** | **SE** | **Group** |
| Black Pyramid | 1.99 | 0.29 | a |
| Yellow Pyramid | 2.44 | 0.34 | a |
| Delta | 0.84 | 0.17 | b |
| Clear Sticky Card | 0.58 | 0.14 | b |
| Yellow Sticky Card | 0.59 | 0.14 | b |
| White Sticky Card | 0.35 | 0.10 | b |
| Blue Sticky Card | 0.43 | 0.11 | b |

**Table S3.** Model-estimated average of green stink bug captures by the single main effects of trap type, and lure type/formulation (single-component: methyl 2-E,4Z-decadienoate; dual-component: murgantiol + methyl E,E,Z-2,4,6-decatrienoate) across full season soybean growth stage (beginning flowering (R1) to harvest). Model-estimated captures represent predicted average counts per sampling event. Mean and SE are on the response (count) scale. Pairwise comparisons used the Sidak adjustment. Levels sharing the same letter are not significantly different (α = 0.05).

| **Trap type** | | | |
| --- | --- | --- | --- |
| **Factor** | **Mean** | **SE** | **Group** |
| Black Pyramid | 1.94 | 0.35 | a |
| Yellow Pyramid | 2.10 | 1.30 | a |
| Delta | 1.33 | 0.26 | ab |
| Clear Sticky Card | 0.80 | 0.19 | bc |
| Yellow Sticky Card | 0.81 | 0.18 | bc |
| White Sticky Card | 0.58 | 0.15 | bc |
| Blue Sticky Card | 0.40 | 0.13 | c |
| **Lure Type/Formulation** | | | |
| **Factor** | **Mean** | **SE** | **Group** |
| Dual | 2.39 | 0.36 | a |
| Single | 0.40 | 0.08 | b |

**Table S4.** Model-estimated average of brown marmorated stink bug captures by the single main effects of trap type, lure type/formulation (single-component: methyl 2-E,4Z-decadienoate; dual-component: murgantiol + methyl E,E,Z-2,4,6-decatrienoate), and year across full season soybean growth stage (beginning flowering (R1) to harvest). Model-estimated captures represent predicted average counts per sampling event. Mean and SE are on the response (count) scale. Pairwise comparisons used the Sidak adjustment. Levels sharing the same letter are not significantly different (α = 0.05).

| **Trap type** | | | |
| --- | --- | --- | --- |
| **Factor** | **Mean** | **SE** | **Group** |
| Black Pyramid | 8.51 | 1.66 | a |
| Yellow Pyramid | 6.62 | 1.22 | a |
| Delta | 1.29 | 0.42 | b |
| Clear Sticky Card | 1.55 | 0.59 | b |
| Yellow Sticky Card | 1.42 | 0.47 | b |
| White Sticky Card | 1.34 | 0.52 | b |
| Blue Sticky Card | 0.98 | 0.51 | b |
| **Lure Type/Formulation** | | | |
| **Factor** | **Mean** | **SE** | **Group** |
| Dual | 12.89 | 2.05 | a |
| Single | 0.36 | 0.11 | b |
| **Year** | | | |
| **Factor** | **Mean** | **SE** | **Group** |
| 2024 | 3.21 | 0.71 | a |
| 2023 | 1.44 | 0.31 | b |

**Table S5.** Model-estimated average of southern green stink bug captures by the single main effects of trap type, and lure type/formulation (single-component: methyl 2-E,4Z-decadienoate; dual-component: murgantiol + methyl E,E,Z-2,4,6-decatrienoate) across full season soybean growth stage (beginning flowering (R1) to harvest). Model-estimated captures represent predicted average counts per sampling event. Mean and SE are on the response (count) scale. Pairwise comparisons used the Sidak adjustment. Levels sharing the same letter are not significantly different (α = 0.05).

| **Trap type** | | | |
| --- | --- | --- | --- |
| **Factor** | **Mean** | **SE** | **Group** |
| Yellow Pyramid | 3.65 | 0.57 | a |
| Black Pyramid | 1.54 | 0.32 | b |
| Delta | 0.93 | 0.22 | bc |
| Clear Sticky Card | 0.63 | 0.20 | bc |
| Yellow Sticky Card | 0.68 | 0.18 | bc |
| White Sticky Card | 0.33 | 0.08 | bc |
| Blue Sticky Card | 0.38 | 0.13 | c |
| **Lure Type/Formulation** | | | |
| **Factor** | **Mean** | **SE** | **Group** |
| Dual | 2.74 | 0.41 | a |
| Single | 0.25 | 0.06 | b |

**Table S6.** Model-estimated average of harlequin bug captures by the single main effects of trap type, lure type/formulation (single-component: methyl 2-E,4Z-decadienoate; dual-component: murgantiol + methyl E,E,Z-2,4,6-decatrienoate), and year across full season soybean growth stage (beginning flowering (R1) to harvest). Model-estimated captures represent predicted average counts per sampling event. Mean and SE are on the response (count) scale. Pairwise comparisons used the Sidak adjustment. Levels sharing the same letter are not significantly different (α = 0.05).

| **Trap type** | | | |
| --- | --- | --- | --- |
| **Factor** | **Mean** | **SE** | **Group** |
| Yellow Sticky Card | 0.53 | 0.11 | a |
| Clear Sticky Card | 0.46 | 0.10 | ab |
| Blue Sticky Card | 0.33 | 0.07 | ab |
| White Sticky Card | 0.28 | 0.06 | b |
| Black Pyramid | 0.11 | 0.03 | c |
| Yellow Pyramid | 0.07 | 0.02 | c |
| Delta | 0.07 | 0.02 | c |
| **Lure Type/Formulation** | | | |
| **Factor** | **Mean** | **SE** | **Group** |
| Dual | 1.23 | 0.14 | a |
| Single | 0.03 | 0.14 | b |
| **Year** | | | |
| **Factor** | **Mean** | **SE** | **Group** |
| 2023 | 0.25 | 0.05 | a |
| 2024 | 0.16 | 0.03 | b |


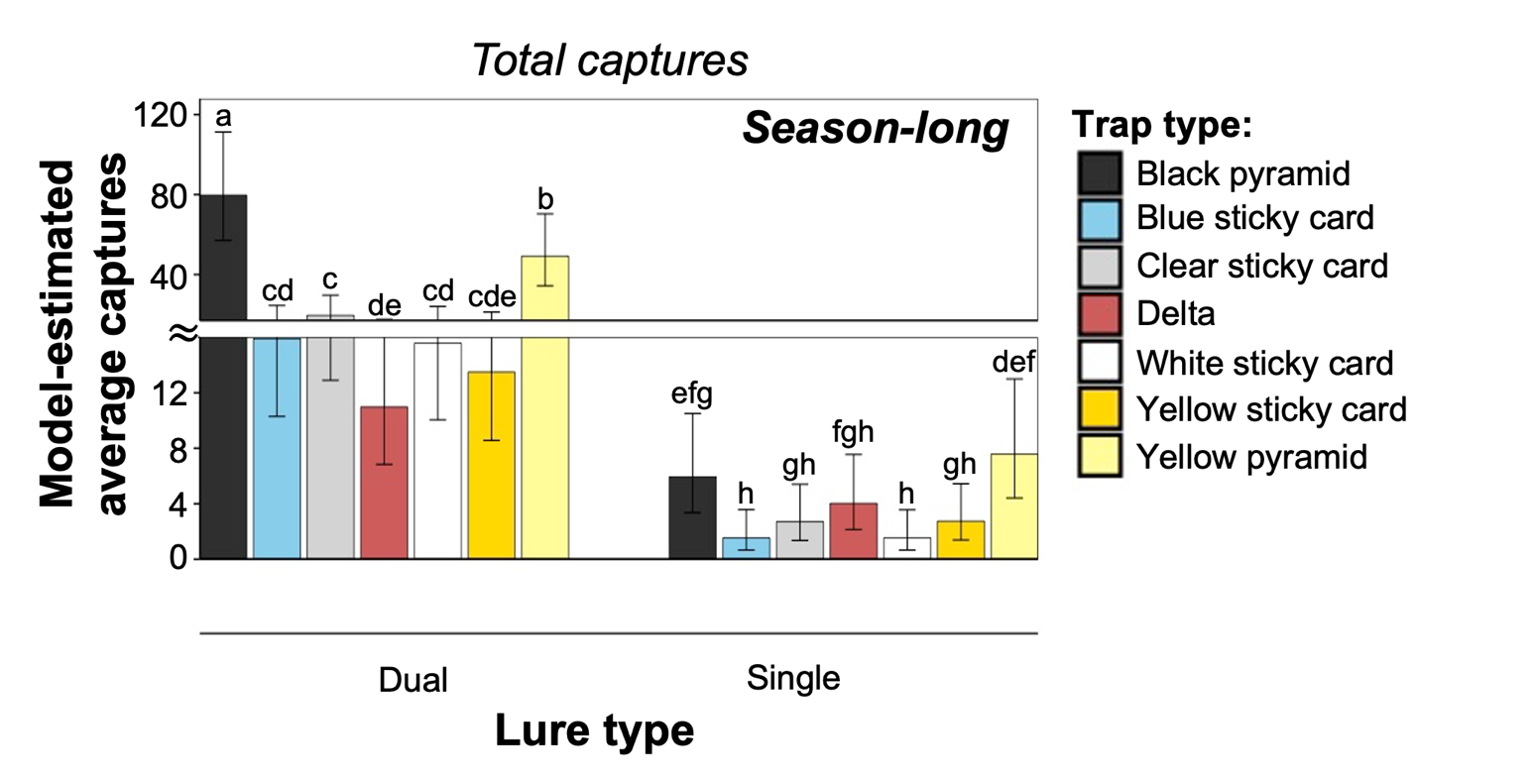


**Figure S1.** Mean (±SE) model-estimated average of total captures (brown stink bug, green stink bug, brown marmorated stink bug, southern green stink bug, harlequin bug, rice stink bug, red-shouldered stink, and spine shouldered stink bug) by combinations of trap type and lure type/formulation (single-component: methyl 2-E,4Z-decadienoate; dual-component: murgantiol + methyl E,E,Z-2,4,6-decatrienoate) across full season soybean growth stage (beginning flowering (R1) to harvest). Model-estimated captures represent predicted average counts per trap-lure combination per sampling event. Error bars represent the 95% confidence interval. Pairwise comparisons used the Sidak test; trap × lure interaction was significant (P < 0.001; Table 1). Bars sharing the same letter within each panel are not significantly different (α = 0.05), based on multiple comparison tests.
