# Supplementary figures and images for: Seasonal dynamics of pheromone traps and lures to monitor stink bugs (Hemiptera: Pentatomidae) in soybean
Source: J Econ Entomol. 2026 May 11;119(3):1919–30. doi: 10.1093/jee/toag125 (PMC13268538; doi:10.1093/jee/toag125)

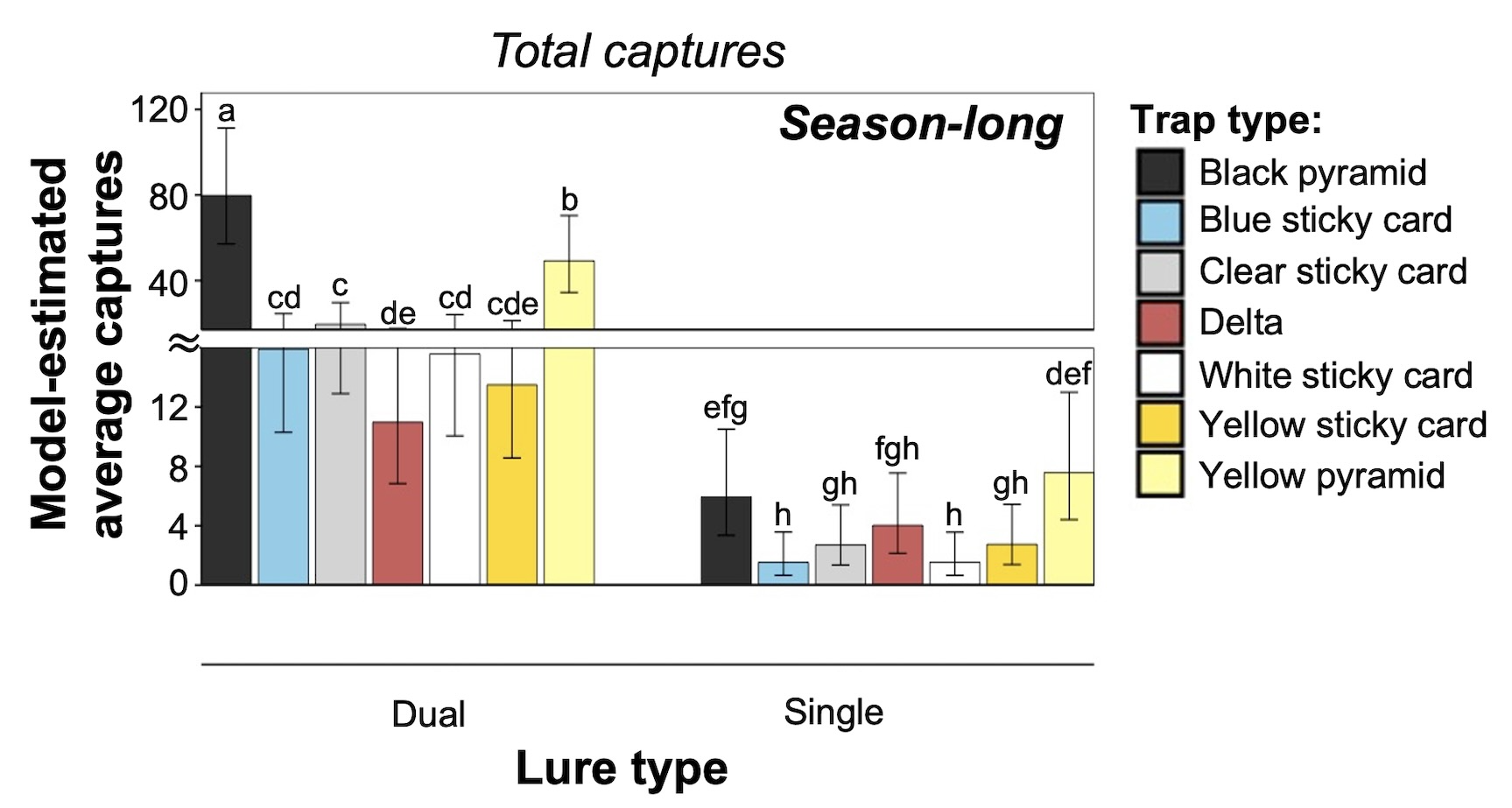

Supplement: toag125_Supplementary_Data [file toag125_supplementary_data.zip › Supplementary_Figure.png]
